# Pentosidine and carboxymethyl-lysine associate differently with prevalent osteoporotic vertebral fracture and various bone markers

**DOI:** 10.1038/s41598-020-78993-w

**Published:** 2020-12-16

**Authors:** Masaki Nakano, Yukio Nakamura, Takako Suzuki, Akiko Miyazaki, Jun Takahashi, Mitsuru Saito, Masataka Shiraki

**Affiliations:** 1 Department of Orthopaedic Surgery, Shinshu University School of Medicine, 3-1-1 Asahi, Matsumoto, Nagano 390-8621 Japan; 2 Department of Human Nutrition, Faculty of Human Nutrition, Tokyo Kasei Gakuin University, 22 Sanban-cho, Chiyoda-ku, Tokyo, 102-8341 Japan; 3 Department of Orthopaedic Surgery, Jikei University School of Medicine, 3-19-18 Nishi-Shimbashi, Minato-ku, Tokyo, 105-8471 Japan; 4 Research Institute and Practice for Involutional Diseases, 1610-1 Meisei, Misato, Azumino, Nagano, 399-8101 Japan

**Keywords:** Metabolic disorders, Endocrinology

## Abstract

Pentosidine (PEN) and carboxymethyl-lysine (CML) are well-recognized advanced glycation end products (AGEs). However, how these AGEs affect the pathophysiology of osteoporosis and osteoporotic fractures remains controversial. This cross-sectional study aimed to investigate the associations of PEN and CML with bone markers, bone mineral density (BMD), and osteoporotic fractures in postmenopausal women from the Nagano Cohort Study. A total of 444 Japanese postmenopausal outpatients (mean ± standard deviation age: 69.8 ± 10.2 years) were enrolled after the exclusion of patients with acute or severe illness or secondary osteoporosis. The relationships among urinary PEN and serum CML levels, various bone markers, lumbar and hip BMD, and prevalent vertebral and long-bone fractures were evaluated. PEN associated significantly with prevalent vertebral fracture after adjustment for other confounders (odds ratio [OR] 1.59, 95% confidence interval [CI] 1.22–2.07; *P* < 0.001), but not with lumbar BMD. In contrast, a significant negative correlation was found between CML and lumbar BMD (*r* =  − 0.180; *P* < 0.001), and this relationship was significant after adjustment for confounders (OR 0.84, 95% CI 0.76–0.93; *P* < 0.01). Although patients with prevalent vertebral fracture had significantly higher CML levels, the association between CML and prevalent vertebral fracture did not reach significance in the multivariate regression model. Both PEN and CML may play important roles in bone health for postmenopausal women, possibly via different mechanisms.

## Introduction

Lifestyle-related metabolic imbalances, such as hyperglycemia, hyperlipidemia, and carbonyl- or oxidative-stress, cause the accumulation of advanced glycation end products (AGEs) through non-enzymatic glycation (i.e., the Maillard reaction)^1,2^. AGEs have been shown to exacerbate renal dysfunction, atherosclerosis, and osteoporosis^3–6^. Once AGEs are formed in bone tissue, they contribute to bone vulnerability via the deterioration of bone matrix proteins, especially collagen. The AGEs in collagen increase with age and have been found to diminish the mechanical properties of bone^7^. The apoptosis of osteocytes is also reportedly promoted by AGEs^8,9^.

Pentosidine (PEN) and carboxymethyl-lysine (CML) are major and well-recognized AGEs that contain arginine or lysine residues, both of which are present in collagen fibers as AGE precursor substances. PEN and CML are non-enzymatically produced in collagen as crosslinked and non-crosslinked types of AGEs, respectively. In contrast to enzymatic crosslinking, an increase in non-enzymatic crosslinking by PEN deteriorates bone strength^10^; urinary PEN excretion increases with age and predicts fractures in men^11^, women^6,12,13^, and diabetic patients^14^. Thus, PEN may be a risk factor for the occurrence of fractures that is independent of other traditional fracture risks, such as age, bone mineral density (BMD), and pre-existing fractures.

Although PEN and total AGEs in bone tissue exist in a proportional relationship, the absolute amount of PEN is smaller than that of other types of AGEs. As a measurable AGE in bone, CML reportedly increases with age and is correlated with previously established measures of bone toughness, including PEN^15^. Since the amount of CML in bone tissue is approximately 100 times higher than that of PEN^15^, CML evaluation may facilitate the development of new diagnostic assays to assess future fracture risk. Moreover, circulating CML levels have already been associated with the risk of hip fractures^16,17^. However, there have been no reports regarding the relationship between CML and vertebral fractures.

There are currently no reports investigating the implications of both PEN and CML on bone metabolism, BMD, and fractures in the same patients. In addition, no studies to uncover why CML associates with fractures have been attempted. The precise mechanisms of PEN and CML in fracture occurrence are therefore unclear and remain controversial. Accordingly, we examined the levels of urinary PEN excretion and serum CML in a cohort of postmenopausal outpatients to assess and compare the impacts of those AGEs on bone status and prevalent osteoporotic fractures in this cross-sectional study.

PEN is conventionally assayed by reversed-phase high-performance liquid chromatography (HPLC) after pre-treatment with heat hydrolysis of the samples^12^; however, this procedure introduces possible artificial error due to differences in the treatment process. We recently developed an enzyme-linked immunosorbent assay (ELISA) system without the need for pre-treatment heat hydrolysis of samples^6,18^. This novel ELISA method was employed to measure urinary PEN levels in the present study.

## Methods

The study protocol of this investigation was reviewed by the ethics committee of the Research Institute and Practice for Involutional Diseases, Japan, in 1993 for the 1st version and in 2000 for the present version, and was carried out in accordance with the principles of the Declaration of Helsinki. Comprehensive written informed consent was provided by all patients enrolled in this study.

### Study subjects

The ongoing Nagano Cohort Study was initiated in 1993 to evaluate ambulatory patients recruited at a primary care institution in Nagano Prefecture, Japan^6,19^. Patients with acute or severe illness, such as acute coronary artery disease, acute cerebrovascular disease, heart failure, respiratory distress, uncontrollable diabetes mellitus (DM) with ≥ 9.0% hemoglobin A_1c_ (HbA1c), end-stage renal failure (estimated glomerular filtration rate [eGFR] < 30 mL/min/1.73m^2^), or terminal cancer, or secondary osteoporosis (e.g., steroid use or primary hyperparathyroidism) were excluded from the study. Patients under any kind of treatment for osteoporosis were included, with exclusion of subjects suffering a major osteoporotic fracture within the previous 6 months. Traumatic fracture patients were also excluded from the study. In total, 446 Japanese postmenopausal participants from the Nagano Cohort Study were included in the present investigation. The proportions of current smokers and drinkers among the participants were low, i.e., 2.1% were smokers and 7.7% were drinkers. After the exclusion of 2 premenopausal cases, the remaining 444 patients were subjected to further analysis. The cohort included patients with DM, dyslipidemia, and hypertension. The condition of the subjects was stable, and all specimens were collected during treatment. The data evaluated in this study were based on the patient characteristics at registration.

### Data collection of patient characteristics

Body height, weight, and trunk circumference were measured by standard procedures, and body mass index (BMI; kg/m^2^) was calculated. Dual energy X-ray absorptiometry (DXA; PRODIGY, GE Healthcare Lunar, Madison, WI) was employed to determine BMD at the lumbar spine and hips as well as trunk and hip fat proportions. The regions of interest for lumbar and hip BMD were the L2–4 and total hip regions, respectively. Assay quality assurance tests were carried out by daily measurements of phantom bone prior to patient examination to avoid machine drift. Routine biochemical data were obtained by respective in-house laboratories. Urinary PEN level was determined by an ELISA method^6,18^ without the need for sample pre-treatment with heat hydrolysis; this method was preferred over PEN measurement in serum samples, which would have required heat hydrolysis and possible inclusion of artificial error. The assay performance of this ELISA system for urinary PEN was reported previously^18^. The sensitivity of the assay system was ≥ 6.24 pmol/mL, and the intra- and inter-assay variations in human urine samples were less than 5% and 4%, respectively. The correlation coefficient between urinary PEN levels measured by HPLC and the ELISA system was 0.815. Serum CML was measured using an ELISA kit (CircuLex, Nagano, Japan)^20^ without the need for heat hydrolysis pre-treatment. The sensitivity of the ELISA kit for CML was ≥ 0.063 µg/mL, and the intra- and inter-assay variances in human serum samples were 5.2–7.4% and 4.7–15.2%, respectively. Neither assay system showed detectable cross-reactivity to non-targeted compounds related to each target, indicating high sensitivities and sufficient specificities.

Regarding bone markers, serum or urinary levels of homocysteine, cross-linked N-telopeptide of type I collagen (NTx), intact osteocalcin, sclerostin, fibroblast growth factor 23 (FGF23), leptin, and adiponectin were measured at LSI Medience (Tokyo, Japan). The diagnoses of DM, dyslipidemia, and hypertension in this study were based on respective guidelines^21–23^. Briefly, DM was diagnosed if HbA1c level was ≥ 6.5% or if the patient was being actively treated for DM. Dyslipidemia was defined as low-density lipoprotein-cholesterol ≥ 140 mg/dL, high-density lipoprotein-cholesterol < 40 mg/dL, or postprandial triglycerides ≥ 200 mg/dL. Hypertension was diagnosed when systolic blood pressure was persistently > 140 mmHg, diastolic pressure was persistently > 90 mmHg, or anti-hypertensive drugs were used.

### Fracture and osteoporosis diagnosis

Prevalent fractures were identified as non-traumatic fractures in the vertebrae, ribs, pelvis, proximal end of the humerus or femur, distal end of the radius, or proximal or distal ends of the tibia or fibula. In the present study, patients were inquired about their medical history of long-bone fracture during interviews. Prevalent vertebral fracture was diagnosed by semi-quantitative analysis of baseline X-ray films of the thoracic and lumbar vertebrae (T4–L4) using an established method^24^ that ranged from grade 0 (normal) to grade 3. Grade 1 was a mild deformity with 10–20% reduction in vertebral body area. Grades 2 and 3 were classified as a moderate deformity (20–40% reduction in vertebral body area) and severe deformity (> 40% reduction in vertebral body area), respectively. In the present study, patients with grade 1 or higher deformities (i.e., more than 10% reduction in vertebral body area) were judged as having prevalent vertebral fracture. Patients with fragility fractures in the vertebrae or proximal end of the femur, other fragility fractures with BMD < 80% of the young adult mean (YAM), or BMD ≤ 70% or − 2.5 standard deviation (SD) of the YAM were diagnosed as having osteoporosis^25^.

### Statistical analysis

Numerical data were presented as mean ± SD and median values. For categorical data, the number and proportion of patients were reported. Data collection included the medical records of 444 Japanese postmenopausal outpatients. We calculated Pearson's product-moment correlation coefficients between log-transformed values of PEN or CML levels and various other characteristic parameters, as well as *P*-values by test for no correlation. The significance of PEN and CML levels according to the presence of co-morbidities and prevalent fractures was evaluated by Student's *t*-test. Moreover, we conducted multivariate analysis to investigate the implications of PEN and CML on prevalent vertebral fracture and BMD of the lumbar spine. To examine the independent associations of PEN and CML with prevalent vertebral fracture and lumbar BMD, we employed the parameters that showed significant correlations with each AGE as confounders in regression analysis; hence, the confounders in the PEN and CML analysis models were not identical. The analysis model for PEN was adjusted for confounders including patient age, body height, lumbar BMD, eGFR, homocysteine, NTx, prevalence of hypertension, and HbA1c or prevalence of DM. For CML, confounder adjustment included patient age, body height, hip fat, lumbar BMD, albumin, blood urea nitrogen (BUN), sclerostin, prevalence of hypertension, and HbA1c or prevalence of DM.

The relationship between DM and higher fragility fracture risk has been well documented^26,27^. Including PEN and CML, AGE formation is reportedly accelerated in DM patients due to an increased concentration of circulating glucose and oxidative stress^28,29^. Bone strength is measured based on BMD and bone quality. We earlier reported an association of PEN with fracture occurrence possibly via deteriorated bone quality by non-enzymatic collagen crosslinking^12,30^. AGEs may also accumulate with other contributing factors, such as aging and eating habits. To examine the independent associations of each AGE with prevalent vertebral fracture and lumbar BMD, we performed multiple regression analysis with adjustment for confounders including DM as well as HbA1c. A two-tailed *P*-value of < 0.05 was considered significantly different for all analyses. All statistical tests were performed using R version 3.6.0 software (https://www.r-project.org/)^31^.

## Results

### Characteristics of study subjects

A total of 444 postmenopausal patients (mean ± SD age: 69.8 ± 10.2 years) fulfilled the selection criteria of the current study. The numerical and categorical characteristics of the patients are summarized in Table 1. In total, 102 patients (23.0%) had been diagnosed as having osteoporosis and were treated with bisphosphonates (*n* = 56), selective estrogen receptor modulators (*n* = 36), daily or weekly preparations of teriparatide (*n* = 4), eldecalcitol (*n* = 3), denosumab (*n* = 2), or estradiol (*n* = 1). No significant differences in PEN or CML levels were noted among patients treated with those therapeutic medications. The number of patients with prevalent long-bone fracture was 33 (7.4%) and that of patients with prevalent vertebral fracture was 106 (23.9%). The mean values of urinary PEN and serum CML in the whole population of the present study were 33.5 ± 11.2 pmol/mgCre and 1.6 ± 0.7 µg/mL, respectively. Since the participants were ambulatory patients who visited a primary care institution, most of them had co-morbidities including DM (*n* = 89; 20.0%), dyslipidemia (*n* = 214; 48.2%), and hypertension (*n* = 220; 49.5%), although the status of all co-morbidities was stable. This study did not include treatment-naïve patients for these co-morbidities.

**Table 1:** Characteristics of study patients.

	Number	Mean ± SD	Median
Age, years	444	69.8 ± 10.2	70.0
Body height, cm	444	151.9 ± 6.3	152.0
Body weight, kg	444	51.5 ± 9.1	50.2
BMI, kg/m^2^	444	22.3 ± 3.5	21.8
Trunk circumference, cm	430	84.2 ± 9.4	84.0
Trunk fat, %	439	27.4 ± 10.6	27.6
Hip fat, %	436	23.7 ± 5.0	23.5
Lumbar BMD, g/cm^2^	444	0.96 ± 0.19	0.93
T-score for lumbar BMD, SD	444	− 1.59 ± 1.30	− 1.81
Hip BMD, g/cm^2^	438	0.77 ± 0.12	0.77
T-score for hip BMD, SD	438	− 1.33 ± 1.03	− 1.40
Albumin, g/dL	411	4.2 ± 0.3	4.3
BUN, mg/dL	424	16.1 ± 4.2	16.0
Creatinine, mg/dL	444	0.64 ± 0.13	0.60
Uric acid, mg/dL	439	4.5 ± 1.2	4.5
eGFR, mL/min/1.73m^2^	444	72.8 ± 16.1	72.6
Total cholesterol, mg/dL	443	209.2 ± 35.2	208.0
Triglycerides^a^, mg/dL	443	146.3 ± 92.9	121.5
HbA1c, %	443	5.6 ± 0.8	5.4
Homocysteine, nmol/mL	433	8.9 ± 3.0	8.3
Folic acid, ng/mL	429	9.7 ± 4.2	8.9
NTx, nmolBCE/mmolCre	439	51.3 ± 27.2	45.7
Osteocalcin, ng/mL	394	7.1 ± 2.8	6.6
Sclerostin, pmol/L	397	36.0 ± 14.8	33.0
FGF23, pg/mL	391	40.7 ± 14.6	38.9
Leptin, ng/mL	396	10.3 ± 8.9	8.0
Adiponectin, µg/mL	390	15.3 ± 8.2	13.2
Calcium, mg/dL	441	9.4 ± 0.5	9.4
Inorganic phosphorus, mg/dL	441	3.5 ± 0.5	3.5
25-OH vitamin D, ng/mL	412	20.6 ± 6.1	20.0
PTH, pg/mL	424	42.7 ± 16.7	40.0
PEN, pmol/mgCre	444	33.5 ± 11.2	32.2
CML, µg/mL	442	1.6 ± 0.7	1.6
Diabetes mellitus, yes	89 (20.0%)		
Dyslipidemia, yes	214 (48.2%)		
Hypertension, yes	220 (49.5%)		
Prevalent long-bone fracture, yes	33 (7.4%)		
Prevalent vertebral fracture, yes	106 (23.9%)		

*SD* standard deviation, *BMI* body mass index, *BMD* bone mineral density, *BUN* blood urea nitrogen, *eGFR* estimated glomerular filtration rate, *HbA1c* hemoglobin A_1c_, *NTx* cross-linked N-telopeptide of type I collagen, *FGF23* fibroblast growth factor 23, *PTH* parathyroid hormone, *PEN* pentosidine, *CML* carboxymethyl-lysine.

^a^Triglycerides were measured with the patient in a postprandial state.

### Associations among AGEs and characteristic parameters

**Figure 1:**
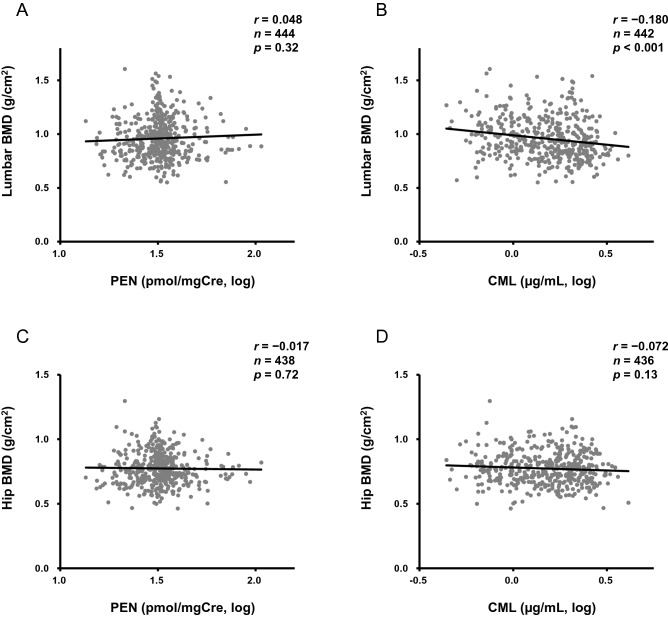
Correlations between AGEs and BMD. The correlation coefficients of (**A**) urinary PEN and (**B**) serum CML for lumbar BMD were 0.048 (*P* = 0.32) and − 0.180 (*P* < 0.001), respectively, and those of (**C**) PEN and (**D**) CML for hip BMD were − 0.017 (*P* = 0.72) and − 0.072 (*P* = 0.13), respectively. *AGEs* advanced glycation end products, *BMD* bone mineral density, *PEN* pentosidine, *CML* carboxymethyl-lysine.

As shown in Table 2, both PEN and CML increased with age. Age might have been a common factor with respect to significant associations with body height. PEN level was significantly associated with trunk circumference, uric acid, eGFR, HbA1c, homocysteine, and NTx. On the other hand, CML had significant correlations with hip fat, albumin, BUN, HbA1c, sclerostin, and FGF23 levels. Of note, since there have been no reports on the association between CML and BMD, this study is the first to reveal a significant negative correlation for CML and lumbar BMD (*r* =  − 0.180; *P* < 0.001), with none detected for PEN and BMD (Table 2 and Fig. 1). There were no associations among parameters involved in calcium metabolism, such as serum calcium, inorganic phosphorus, 25-OH vitamin D, or parathyroid hormone, with PEN or CML (data not shown); thus, the influence of AGEs on calcium metabolism might be less predominant. On the other hand, HbA1c level showed significant positive correlations with both PEN and CML, suggesting that glucose metabolism was a notable determinant for the AGEs.

**Table 2:** Associations of AGEs with patient characteristics.

	PEN (pmol/mgCre, log)		CML (µg/mL, log)	
	Correlation coefficient	*P* value	Correlation coefficient	*P* value
Age, years	0.230	< 0.001	0.212	< 0.001
Body height, cm	− 0.138	< 0.01	− 0.145	< 0.01
Body weight, kg	0.053	0.27	− 0.041	0.39
Trunk circumference, cm	0.156	< 0.01	0.077	0.11
Trunk fat, %	0.031	0.52	0.059	0.22
Hip fat, %	0.024	0.61	0.117	< 0.05
Lumbar BMD, g/cm^2^	0.048	0.32	− 0.180	< 0.001
Hip BMD, g/cm^2^	− 0.017	0.72	− 0.072	0.13
Albumin, g/dL	0.016	0.74	− 0.100	< 0.05
BUN, mg/dL	0.064	0.19	0.131	< 0.01
Creatinine, mg/dL	0.078	0.099	0.030	0.54
Uric acid, mg/dL	0.134	< 0.01	0.018	0.71
eGFR, mL/min/1.73m^2^	− 0.097	< 0.05	− 0.080	0.094
Total cholesterol, mg/dL	− 0.061	0.20	0.064	0.18
Triglycerides, mg/dL	0.014	0.78	0.044	0.36
HbA1c, %	0.146	< 0.01	0.171	< 0.001
Homocysteine, nmol/mL, log	0.160	< 0.001	0.019	0.69
NTx, nmolBCE/mmolCre, log	0.145	< 0.01	− 0.014	0.77
Osteocalcin, ng/mL, log	− 0.044	0.38	0.048	0.34
Sclerostin, pmol/L, log	0.079	0.12	0.132	< 0.01
FGF23, pg/mL, log	0.062	0.22	0.105	< 0.05
Leptin, ng/mL, log	0.015	0.77	0.046	0.36
Adiponectin, µg/mL, log	0.063	0.21	0.045	0.38

*AGEs* advanced glycation end products, *PEN* pentosidine, *CML* carboxymethyl-lysine, *BMD* bone mineral density, *BUN* blood urea nitrogen, *eGFR* estimated glomerular filtration rate, *HbA1c* hemoglobin A_1c_, *NTx* cross-linked N-telopeptide of type I collagen, *FGF23* fibroblast growth factor 23.

### Associations of AGEs with co-morbidities, prevalent osteoporotic fracture, and BMD

**Figure 2:**
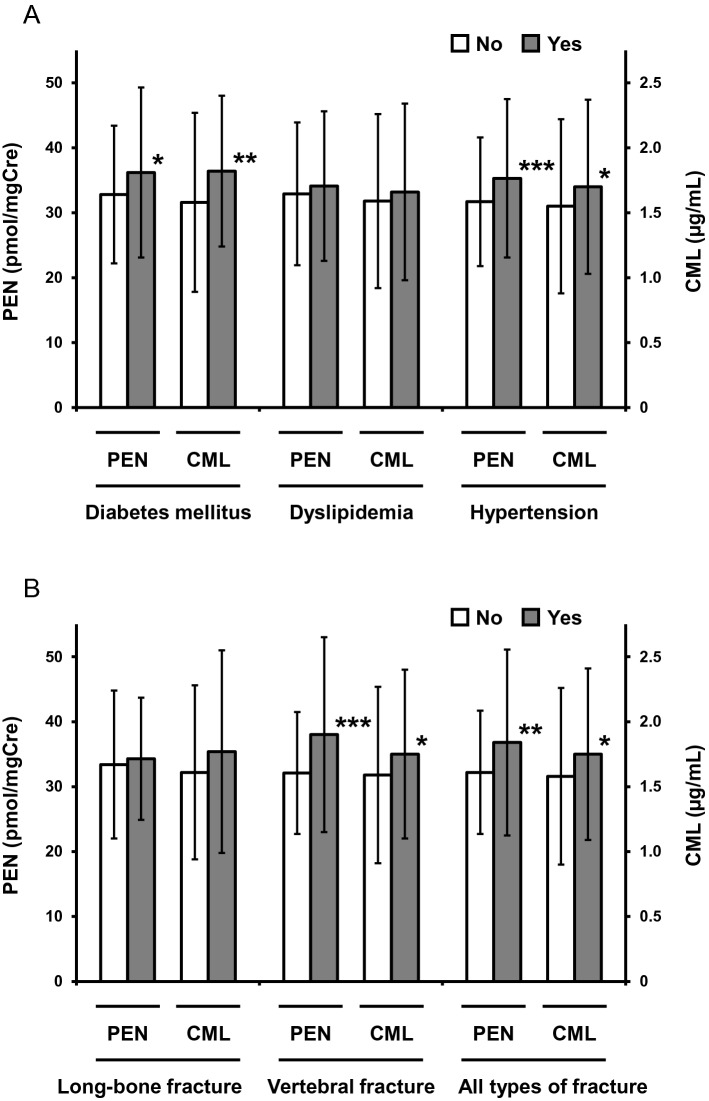
AGE levels according to the presence of co-morbidities and prevalent fractures. Mean ± SD values of PEN (left axis) and CML (right axis) in patients with or without (**A**) co-morbidities and (**B**) prevalent fractures are presented. Asterisks indicate significant differences vs. patients without each disorder evaluated by Student's *t*-test (*P < 0.05, **P < 0.01, and ***P < 0.001). *AGE* advanced glycation end product, *SD* standard deviation, *PEN* pentosidine, *CML* carboxymethyl-lysine.

**Figure 3:**
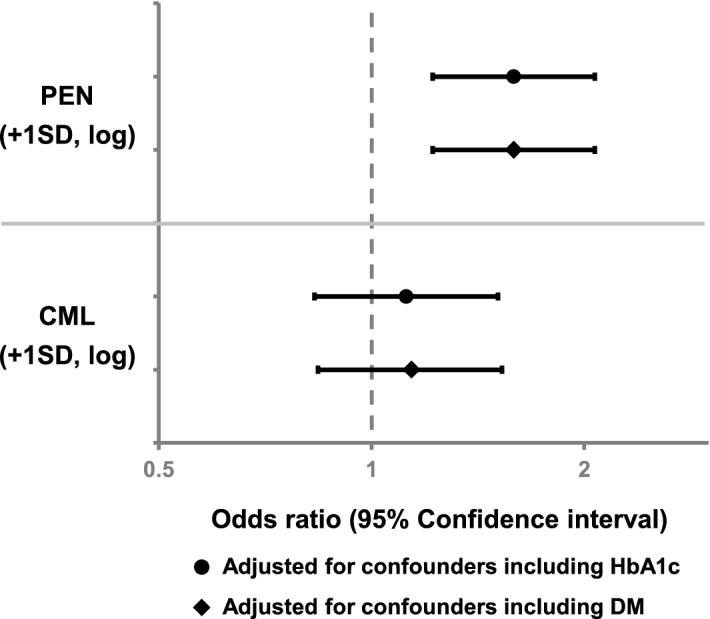
Multiple logistic regression analysis for prevalent vertebral fracture by AGE level. PEN was adjusted for patient age, body height, lumbar BMD, eGFR, homocysteine, NTx, prevalence of hypertension, and HbA1c or prevalence of DM. CML was adjusted for patient age, body height, hip fat, lumbar BMD, albumin, BUN, sclerostin, prevalence of hypertension, and HbA1c or prevalence of DM. *AGE* advanced glycation end product, *PEN* pentosidine, *BMD* bone mineral density, *eGFR* estimated glomerular filtration rate, *NTx* cross-linked N-telopeptide of type I collagen, *HbA1c* hemoglobin A_1c_, *DM* diabetes mellitus, *CML* carboxymethyl-lysine, *BUN* blood urea nitrogen, *SD* standard deviation.

In regard to relations between AGEs and co-morbidities, significantly higher levels of both PEN and CML were observed in the presence of DM and hypertension, but not dyslipidemia (Fig. 2A and Supplementary Table 1). On the other hand, patients with prevalent vertebral fracture, but not long-bone fracture, showed significantly higher urinary PEN (*P* < 0.001) and serum CML (*P* < 0.05) levels as compared with patients without osteoporotic vertebral fracture (Fig. 2B and Supplementary Table 1). Furthermore, PEN values exhibited a significantly independent association with prevalent vertebral fracture after adjustment for confounders (odds ratio [OR] 1.59, 95% confidence interval [CI] 1.22–2.07; *P* < 0.001) (Fig. 3 and Supplementary Table 2). The relationship between PEN and vertebral fracture was consistent with our previous observations^12,13^. However, an independent association of CML with osteoporotic vertebral fracture prevalence was absent in multiple logistic regression analysis after adjustment for confounders (Fig. 3 and Supplementary Table 3).

**Figure 4:**
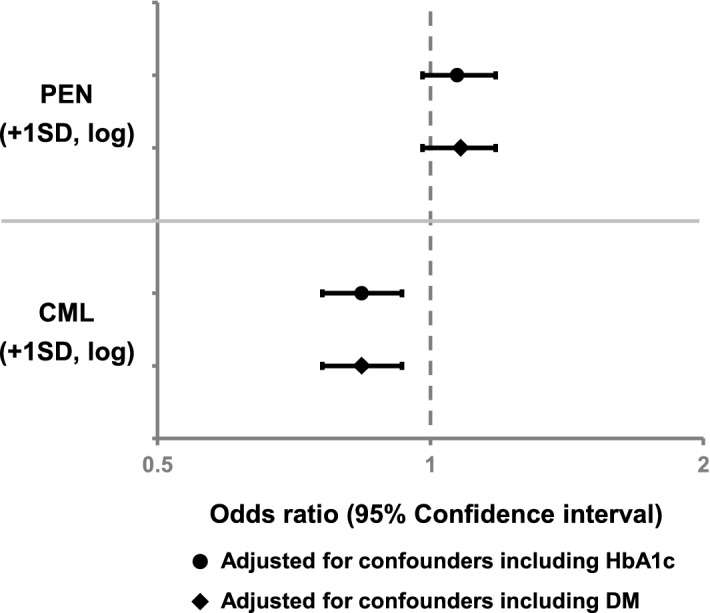
Multiple regression analysis for lumbar BMD by AGE level. PEN was adjusted for patient age, body height, eGFR, homocysteine, NTx, prevalence of hypertension, and HbA1c or prevalence of DM. CML was adjusted for patient age, body height, hip fat, albumin, BUN, sclerostin, prevalence of hypertension, and HbA1c or prevalence of DM. *BMD* bone mineral density, *AGE* advanced glycation end product, *PEN* pentosidine, *eGFR* estimated glomerular filtration rate, *NTx* cross-linked N-telopeptide of type I collagen, *HbA1c* hemoglobin A_1c_, *DM* diabetes mellitus, *CML* carboxymethyl-lysine, *BUN* blood urea nitrogen, *SD* standard deviation.

We subsequently examined the relationships between the AGEs and BMD. PEN failed to demonstrate a significant association with lumbar BMD in multiple regression analysis (Fig. 4 and Supplementary Table 4), whereas CML values showed a significant negative independent association with lumbar BMD after adjustment for confounders (OR 0.84, 95% CI 0.76–0.93; *P* < 0.01) (Fig. 4 and Supplementary Table 5). As shown in Table 2, urinary PEN and NTx had a significant positive correlation (*r* = 0.145; *P* < 0.01). However, PEN did not demonstrate a significantly independent association with lumbar BMD, while the significance of NTx remained after adjustment for confounders (Supplementary Table 4). These observations suggest that higher urinary PEN excretion may be linked to increased bone resorption. On the other hand, a significant correlation between serum CML and sclerostin level (*r* = 0.132; *P* < 0.01) was observed (Table 2). As presented in Supplementary Table 5, both CML and sclerostin were significantly associated with lumbar BMD after adjustment for confounders, indicating that these parameters had independent relationships with BMD of the lumbar spine.

## Discussion

In the present study, we cross-sectionally investigated for associations of the AGEs PEN and CML with prevalent osteoporotic fractures and bone status together with various metabolic parameters. PEN associated significantly with prevalent vertebral fracture, with no correlation with BMD. In contrast, CML correlated negatively with lumbar BMD, with no independent relationship with prevalent vertebral fracture. Since we observed no significant relationship between PEN, a crosslinked type of AGE, and BMD, it would appear that PEN associated independently with the occurrence of fracture via collagen network deterioration, leading to impaired bone quality without affecting BMD. On the other hand, as a non-crosslinked type of AGE, CML exhibited significant positive correlations with serum levels of sclerostin and FGF23, both of which secreted by osteocytes to impair bone mineralization. Thus, CML could have affected osteocytes within the bone matrix and contributed to fracture occurrence through lowered BMD. Both AGEs therefore appear to impact bone status and health, although possibly through different mechanisms.

With regard to glucose homeostasis, both PEN and CML displayed a positive significant relationship with HbA1c, a glycated hemoglobin. Hanssen et al. have reported no significant associations for AGEs with glucose metabolism^32^, while conflicting results exist for AGEs and insulin secretion or resistance^33,34^. Semba et al. described that serum CML concentration was strongly and inversely affected by body fat^35^, possibly since it was preferentially deposited in fat tissue or because adipocytes influenced the metabolism of AGEs. In contrast, our findings showed a significant positive correlation between serum CML level and hip fat mass as well as the prevalence rate of DM. Liman et al. very recently reported in an Indonesian cohort that CML levels might be associated with obesity, which was in agreement with our findings, and advised a reduction in CML-rich foods to reduce obesity risk^36^. The discrepancy between Semba's data and our own might be attributable to differences in patient ethnicity (Caucasian vs. Japanese or Asian) or the composition of co-morbidities, especially DM.

In this study, both PEN and CML values demonstrated significant associations with prevalent vertebral fracture. However, the mechanisms of long-bone and vertebral fracture occurrence appeared to differ. Our findings revealed that while PEN had no correlation with BMD, CML exhibited a significant negative relationship. CML has been reported to accelerate the apoptosis of osteoblastic cells and impair osteocyte functions^8^. Since CML is a non-crosslinked type of AGEs, its impact on bone matrix is thought to be relatively small. Hence, CML may affect the occurrence of fractures by the impairment of osteoblast or osteocyte functions, leading to reduced BMD. Indeed, serum sclerostin and FGF23 levels were positively correlated with CML, suggesting the involvement of osteocytes within the bone matrix. As the associations of CML and sclerostin with lumbar BMD were independent of each other, the implications of CML on osteocyte function remain controversial. Meanwhile, as a crosslinked type of AGEs, PEN seems to work not as a cytotoxin, but rather as a strong contributing factor to age-related changes in the collagen network and bone quality^37^. As there was no association between PEN and BMD in the current study, PEN might be related to fractures via bone matrix vulnerability.

The present study demonstrated an association of PEN, but not CML, with homocysteine level. Homocysteine generally increases with insufficient vitamin B group levels and is an independent risk factor for osteoporotic fractures^38–40^, which is similar to PEN. Imbalanced glucose metabolism and subsequent diabetes cause incremental vitamin B consumption to produce a relative vitamin B deficiency^41^. We earlier reported a relationship between higher circulating homocysteine level and PEN accumulation^42,43^ suggesting the association of a vitamin B deficiency caused by abnormal glucose metabolism with osteoporotic fracture risk. Indeed, homocysteine could be a susceptibility factor for fractures^44^. It is noteworthy that not only a vitamin B group deficiency causing abnormal collagen conditions, but also imbalanced bone metabolism brought about by abnormalities in osteocyte function (e.g., sclerostin and FGF23) may be a prominent fracture-related factor as well. Since osteocytes exist within the collagen network, collagen aberrations should presumably affect osteocytes. However, in this study, no associations were found between PEN and sclerostin or FGF23 levels. Therefore, the crosslinked AGE PEN was believed to relate to fracture occurrence through the deterioration of bone quality without affecting osteocyte function and subsequently lowered BMD. Those metabolic abnormalities due to impaired osteocyte function could have also been provoked by AGEs other than PEN, possibly by a non-crosslinked AGE such as CML.

Patients with type 2 DM have an increased risk of bone fracture irrespectively of normal or high BMD^45,46^. Altered bone microarchitecture and/or poor bone quality have been suggested as possible causes of such fractures^47^. There have been contradictory reports on the association of circulating CML levels with the risk of hip fracture. Barzilay et al. identified an association between increasing CML levels and hip fracture risk that was independent of hip BMD^16^, whereas Lamb et al. showed that men with lower quartiles of plasma CML had a higher incidence of hip fracture^17^. On the other hand, there have been no reports on the relationship between CML and vertebral fracture, with conflicting results also existing on the association between circulating CML levels and obesity^35,36^. The present study revealed significantly higher serum CML levels in patients with DM or prevalent vertebral fracture. Although CML did not independently associate with the occurrence of vertebral fracture, it correlated negatively with lumbar BMD. CML has been reported to accelerate the apoptosis of osteoblastic cells and hamper osteocyte functions^8^. Indeed, our results showed significant positive correlations for CML with serum levels of sclerostin and FGF23, both of which secreted by osteocytes to impair bone mineralization. Therefore, CML is presumed to affect osteocytes within the bone matrix and relate to vertebral fracture occurrence through lowered BMD. Our multivariate regression model adjusted for confounders including PEN or CML showed no significant independent associations of DM with prevalent vertebral fracture and lumbar BMD. Thus, the pathophysiology of diabetic fractures may be due to elevated levels of PEN, CML, or other AGEs affecting bone fragility or BMD.

It is well known that since PEN and CML are AGEs, these protein degradation factors accumulate with age. In addition to AGEs, other fracture-related factors increase over time, such as homocysteine. Thus, the associations of PEN and CML with fractures may reflect either direct or indirect influences via the regulation of fracture-related factors. Based on our findings, the relationships between AGEs and fractures seemed to depend on fracture type, which might have been the result of insufficient statistical power due to the limitation of relatively few cases of long-bone fracture. Re-examination of the Nagano Cohort Study after an increase in long-bone fracture cases is needed for a more detailed analysis of AGEs and fractures. Since we investigated the associations between AGEs levels and prevalent osteoporotic fractures as a cross-sectional study, we could not identify a causal relationship of AGEs with prevalent fractures. The exact time of prevalent fracture occurrence could not be pinpointed, especially for vertebral fracture, due to the lack of clinical episodes. Therefore, the pathophysiological importance of CML in fractures requires confirmation by a prospective study design. In addition, as this study consisted only of postmenopausal women, its conclusions could not be expanded to the general population. Further observational studies containing longitudinal investigation or analyses of community-dwelling participants are required to confirm the associations between AGEs and fractures.

In conclusion, PEN as well as CML were associated with prevalent vertebral fracture in postmenopausal women. The mechanism of PEN might be independent of lumbar BMD, while that of CML could be BMD dependent. Lastly, it is considered that the association between AGEs and fracture occurrence is related to various metabolic abnormalities accompanying age. Prospective interventional studies are needed to clarify whether or not AGEs are the direct cause of osteoporotic fractures.

## Supplementary Information

Supplementary Information

## Data Availability

The data analyzed or generated during the current study are available from the corresponding author on reasonable request.
