# Supplementary material for: Pentosidine and carboxymethyl-lysine associate differently with prevalent osteoporotic vertebral fracture and various bone markers
Source: Sci Rep. 2020 Dec 16;10:22090. doi: 10.1038/s41598-020-78993-w (PMC7744574; doi:10.1038/s41598-020-78993-w)
Supplement: Supplementary file 1 — Supplementary Information [file 41598_2020_78993_MOESM1_ESM.docx]

**Supplementary Table 1a.** PEN and CML levels according to the presence of co-morbidities

| Type of co-morbidity | PEN, pmol/mgCre | | | CML, µg/mL | | |
| --- | --- | --- | --- | --- | --- | --- |
|  | No | Yes | *P*-value | No | Yes | *P*-value |
| Diabetes mellitus | 32.8 ± 10.6 | 36.2 ± 13.1 | < 0.05 | 1.58 ± 0.69 | 1.82 ± 0.58 | < 0.01 |
| Dyslipidemia | 32.9 ± 11.0 | 34.1 ± 11.5 | 0.28 | 1.59 ± 0.67 | 1.66 ± 0.68 | 0.24 |
| Hypertension | 31.7 ± 9.9 | 35.3 ± 12.2 | < 0.001 | 1.55 ± 0.67 | 1.70 ± 0.67 | < 0.05 |

PEN, pentosidine; CML, carboxymethyl-lysine

**Supplementary Table 1b.** PEN and CML levels according to the presence of prevalent fractures

| Type of prevalent fracture | PEN, pmol/mgCre | | | CML, µg/mL | | |
| --- | --- | --- | --- | --- | --- | --- |
|  | No | Yes | *P*-value | No | Yes | *P*-value |
| Long-bone fracture | 33.4 ± 11.4 | 34.3 ± 9.4 | 0.67 | 1.61 ± 0.67 | 1.77 ± 0.78 | 0.19 |
| Vertebral fracture | 32.1 ± 9.4 | 38.0 ± 15.0 | < 0.001 | 1.59 ± 0.68 | 1.75 ± 0.65 | < 0.05 |
| All types of fracture | 32.2 ± 9.5 | 36.8 ± 14.3 | < 0.01 | 1.58 ± 0.68 | 1.75 ± 0.66 | < 0.05 |

PEN, pentosidine; CML, carboxymethyl-lysine

**Supplementary Table 2a.** Multiple logistic regression analysis for prevalent vertebral fracture by PEN level (+1SD, log) adjusted for confounders including HbA1c

|  | Odds ratio | 95% CI | *P*-value |
| --- | --- | --- | --- |
| Age (+1SD) | 1.64 | 1.15–2.34 | < 0.01 |
| Body height (+1SD) | 0.59 | 0.43–0.81 | < 0.01 |
| Lumbar BMD (+1SD) | 0.52 | 0.38–0.71 | < 0.001 |
| eGFR (+1SD) | 0.93 | 0.69–1.25 | 0.63 |
| Homocysteine (+1SD, log) | 0.90 | 0.69–1.17 | 0.41 |
| NTx (+1SD, log) | 0.96 | 0.74–1.24 | 0.75 |
| Hypertension (yes) | 0.87 | 0.51–1.49 | 0.62 |
| HbA1c (+1SD) | 0.88 | 0.67–1.15 | 0.34 |
| PEN (+1SD, log) | 1.59 | 1.22–2.07 | < 0.001 |

PEN, pentosidine; SD, standard deviation; HbA1c, hemoglobin A_1c_; CI, confidence interval; BMD, bone mineral density; eGFR, estimated glomerular filtration rate; NTx, cross-linked N-telopeptide of type I collagen

**Supplementary Table 2b.** Multiple logistic regression analysis for prevalent vertebral fracture by PEN level (+1SD, log) adjusted for confounders including DM

|  | Odds ratio | 95% CI | *P*-value |
| --- | --- | --- | --- |
| Age (+1SD) | 1.62 | 1.14–2.31 | < 0.01 |
| Body height (+1SD) | 0.59 | 0.43–0.81 | < 0.001 |
| Lumbar BMD (+1SD) | 0.51 | 0.37–0.70 | < 0.001 |
| eGFR (+1SD) | 0.92 | 0.68–1.23 | 0.56 |
| Homocysteine (+1SD, log) | 0.88 | 0.68–1.15 | 0.35 |
| NTx (+1SD, log) | 0.95 | 0.73–1.22 | 0.67 |
| Hypertension (yes) | 0.91 | 0.53–1.56 | 0.73 |
| DM (yes) | 0.65 | 0.33–1.28 | 0.21 |
| PEN (+1SD, log) | 1.59 | 1.22–2.07 | < 0.001 |

PEN, pentosidine; SD, standard deviation; DM, diabetes mellitus; CI, confidence interval; BMD, bone mineral density; eGFR, estimated glomerular filtration rate; NTx, cross-linked N-telopeptide of type I collagen

**Supplementary Table 3a.** Multiple logistic regression analysis for prevalent vertebral fracture by CML level (+1SD, log) adjusted for confounders including HbA1c

|  | Odds ratio | 95% CI | *P*-value |
| --- | --- | --- | --- |
| Age (+1SD) | 1.68 | 1.15–2.44 | < 0.01 |
| Body height (+1SD) | 0.56 | 0.40–0.78 | < 0.001 |
| Hip fat (+1SD) | 1.03 | 0.78–1.37 | 0.83 |
| Lumbar BMD (+1SD) | 0.56 | 0.40–0.79 | < 0.001 |
| Albumin (+1SD) | 0.84 | 0.62–1.13 | 0.24 |
| BUN (+1SD) | 1.05 | 0.81–1.36 | 0.73 |
| Sclerostin (+1SD, log) | 1.19 | 0.89–1.59 | 0.24 |
| Hypertension (yes) | 0.83 | 0.46–1.49 | 0.53 |
| HbA1c (+1SD) | 0.97 | 0.74–1.28 | 0.84 |
| CML (+1SD, log) | 1.12 | 0.83–1.51 | 0.45 |

CML, carboxymethyl-lysine; SD, standard deviation; HbA1c, hemoglobin A_1c_; CI, confidence interval; BMD, bone mineral density; BUN, blood urea nitrogen

**Supplementary Table 3b.** Multiple logistic regression analysis for prevalent vertebral fracture by CML level (+1SD, log) adjusted for confounders including DM

|  | Odds ratio | 95% CI | *P*-value |
| --- | --- | --- | --- |
| Age (+1SD) | 1.68 | 1.16–2.45 | < 0.01 |
| Body height (+1SD) | 0.55 | 0.39–0.78 | < 0.001 |
| Hip fat (+1SD) | 1.03 | 0.78–1.37 | 0.82 |
| Lumbar BMD (+1SD) | 0.56 | 0.40–0.79 | < 0.001 |
| Albumin (+1SD) | 0.86 | 0.63–1.18 | 0.35 |
| BUN (+1SD) | 1.04 | 0.80–1.35 | 0.79 |
| Sclerostin (+1SD, log) | 1.20 | 0.90–1.60 | 0.22 |
| Hypertension (yes) | 0.85 | 0.47–1.52 | 0.58 |
| DM (yes) | 0.77 | 0.36–1.65 | 0.50 |
| CML (+1SD, log) | 1.14 | 0.84–1.53 | 0.40 |

CML, carboxymethyl-lysine; SD, standard deviation; DM, diabetes mellitus; CI, confidence interval; BMD, bone mineral density; BUN, blood urea nitrogen

**Supplementary Table 4a.** Multiple regression analysis for lumbar BMD by PEN level (+1SD, log) adjusted for confounders including HbA1c

|  | Odds ratio | 95% CI | *P*-value |
| --- | --- | --- | --- |
| Age (+1SD) | 0.86 | 0.77–0.96 | < 0.05 |
| Body height (+1SD) | 1.28 | 1.15–1.42 | < 0.001 |
| eGFR (+1SD) | 0.95 | 0.86–1.05 | 0.34 |
| Homocysteine (+1SD, log) | 1.11 | 1.01–1.21 | < 0.05 |
| NTx (+1SD, log) | 0.89 | 0.82–0.97 | < 0.05 |
| Hypertension (yes) | 1.34 | 1.12–1.62 | < 0.01 |
| HbA1c (+1SD) | 1.11 | 1.01–1.21 | < 0.05 |
| PEN (+1SD, log) | 1.07 | 0.98–1.18 | 0.13 |

BMD, bone mineral density; PEN, pentosidine; SD, standard deviation; HbA1c, hemoglobin A_1c_; CI, confidence interval; eGFR, estimated glomerular filtration rate; NTx, cross-linked N-telopeptide of type I collagen

**Supplementary Table 4b.** Multiple regression analysis for lumbar BMD by PEN level (+1SD, log) adjusted for confounders including DM

|  | Odds ratio | 95% CI | *P*-value |
| --- | --- | --- | --- |
| Age (+1SD) | 0.87 | 0.78–0.98 | < 0.05 |
| Body height (+1SD) | 1.29 | 1.16–1.43 | < 0.001 |
| eGFR (+1SD) | 0.96 | 0.87–1.06 | 0.46 |
| Homocysteine (+1SD, log) | 1.11 | 1.01–1.22 | < 0.05 |
| NTx (+1SD, log) | 0.90 | 0.82–0.98 | < 0.05 |
| Hypertension (yes) | 1.32 | 1.10–1.60 | < 0.01 |
| DM (yes) | 1.25 | 0.99–1.56 | 0.056 |
| PEN (+1SD, log) | 1.08 | 0.98–1.18 | 0.12 |

BMD, bone mineral density; PEN, pentosidine; SD, standard deviation; DM, diabetes mellitus; CI, confidence interval; eGFR, estimated glomerular filtration rate; NTx, cross-linked N-telopeptide of type I collagen

**Supplementary Table 5a.** Multiple regression analysis for lumbar BMD by CML level (+1SD, log) adjusted for confounders including HbA1c

|  | Odds ratio | 95% CI | *P*-value |
| --- | --- | --- | --- |
| Age (+1SD) | 0.90 | 0.79–1.01 | 0.078 |
| Body height (+1SD) | 1.23 | 1.10–1.37 | < 0.001 |
| Hip fat (+1SD) | 1.08 | 0.98–1.18 | 0.13 |
| Albumin (+1SD) | 1.06 | 0.96–1.17 | 0.26 |
| BUN (+1SD) | 0.94 | 0.86–1.04 | 0.22 |
| Sclerostin (+1SD, log) | 1.20 | 1.09–1.32 | < 0.001 |
| Hypertension (yes) | 1.26 | 1.03–1.54 | < 0.05 |
| HbA1c (+1SD) | 1.09 | 0.99–1.20 | 0.094 |
| CML (+1SD, log) | 0.84 | 0.76–0.93 | < 0.01 |

BMD, bone mineral density; CML, carboxymethyl-lysine; SD, standard deviation; HbA1c, hemoglobin A_1c_; CI, confidence interval; BUN, blood urea nitrogen

**Supplementary Table 5b.** Multiple regression analysis for lumbar BMD by CML level (+1SD, log) adjusted for confounders including DM

|  | Odds ratio | 95% CI | *P*-value |
| --- | --- | --- | --- |
| Age (+1SD) | 0.90 | 0.80–1.02 | 0.090 |
| Body height (+1SD) | 1.23 | 1.10–1.38 | < 0.001 |
| Hip fat (+1SD) | 1.07 | 0.97–1.18 | 0.15 |
| Albumin (+1SD) | 1.05 | 0.95–1.17 | 0.34 |
| BUN (+1SD) | 0.95 | 0.86–1.05 | 0.29 |
| Sclerostin (+1SD, log) | 1.19 | 1.08–1.32 | < 0.001 |
| Hypertension (yes) | 1.25 | 1.02–1.53 | < 0.05 |
| DM (yes) | 1.22 | 0.94–1.57 | 0.14 |
| CML (+1SD, log) | 0.84 | 0.76–0.93 | < 0.01 |

BMD, bone mineral density; CML, carboxymethyl-lysine; SD, standard deviation; DM, diabetes mellitus; CI, confidence interval; BUN, blood urea nitrogen
